# Efficacy of Minimum Application of Chlorfluazuron Baiting to Control Urban Subterranean Termite Populations of *Coptotermes gestroi* (Wasmann) (Blattodea: Rhinotermitidae)

**DOI:** 10.3390/insects11090569

**Published:** 2020-08-25

**Authors:** Wan Ahmad Syahir Wan Umar, Abdul Hafiz Ab Majid

**Affiliations:** Household and Structural Urban Entomology Laboratory, Vector Control Research Unit, School of Biological Sciences, Universiti Sains Malaysia, Minden 11800, Penang, Malaysia; wansyahir@yahoo.com

**Keywords:** chlorfluazuron bait, termite baiting, minimum application, *Coptotermes gestroi*, subterranean termite

## Abstract

**Simple Summary:**

The termite baiting system has emerged as a popular option for controlling subterranean termites. The effectiveness of termite baiting depends on the foraging of the termites to encounter the bait, feed on the bait, and the horizontal transfer of residual insecticide deposits between nestmates. However, termite baiting can be costly and time consuming. Thus, this study looks to minimize the termite baiting application which leads to the total colony elimination. Overall, this study found that a termite colony population can be eliminated by selective termite baiting treatment.

**Abstract:**

Termite infestations in urban areas are a serious problem because they cause negative economic effects, reduce the esthetic value of buildings, damage crops, and require household repairs. Chemical controls are the most common method used against subterranean termites, and baiting has emerged as one of the prominent control methods. The goal of this research was to determine the efficacy of termite baiting by treating one of six active termite stations (selective baiting) with chlorfluazuron baits to eradicate six populations of subterranean termites. This work shows that the placement of chlorfluazuron baits in one of the active stations was sufficient to destroy a colony that was interconnected with multiple chlorfluazuron-free stations. In general, it requires an average of 4–8 weeks for a quantity of less than 300 g of chlorfluazuron bait to remove a termite infestation at the study site.

## 1. Introduction

There are an estimated 3105 species of termite globally [1]. Of approximately 80 subterranean termite pest species, 38 are of the genus *Coptotermes*, which accounts for the largest share, closely followed by the genera *Macrotermes*, *Reticulitermes*, and *Odontotermes* [2].

Numerous means exist of preventing and controlling termite infestations, including physical, chemical, and biological controls. In the past, chemical termiticide has been commonly used by practitioners of termite control [3,4].

Furthermore, chemical controls are the most common method used against pests of urban forests in Malaysia [5], for example, trees infested by *C. gestroi* and *Coptotermes curvignathus* are usually treated with an insecticide such as chlorpyrifos, fipronil, or imidacloprid [6]. However, as Lee et al. (2014) noted, the baiting system has emerged as a popular option for controlling subterranean termites [6]. The effectiveness of baiting depends on the foraging of the termites to encounter the bait, feed on the bait, and the horizontal transfer of residual insecticide deposits between nestmates [7].

The active ingredients used in baiting systems are of low mammalian toxicity, affect only the targeted pest species, and use a relatively small amount of insecticide compared to standard chemical soil treatment [8]. Chlorfluazuron is a member of the benzoylphenylurea (BPU) group that contains a lethal chitin synthesis inhibitor (CSI) which can be applied to several insect orders [9]. CSI causes chitin synthesis in the cuticle of the insect to be disrupted during the molting process [10]. Several studies have successfully documented the use of CSIs in eradicating lower termite species (rhinotermitids) [11,12,13,14,15,16]. According to Chouvenc and Su (2017), as little as 1.1 g of bait matrix (=5.5 mg noviflumuron) fed for a single day was sufficient to eliminate colonies in 90 days [17]. This study shows that only a small fraction of the bait is needed for the elimination of a large laboratory colony [17].

Through the use of chlorfluazuron baits to remove subterranean termite populations, this study aims to evaluate the efficiency of treatment by treating one of a group of active termite stations (selective baiting). The time taken for suppression of the colony and the total baits consumed by the termites to achieve colony elimination were recorded. 

## 2. Materials and Methods

### 2.1. Evaluation of the Effectiveness of Selective Baiting Using Chlorfluazuron to Control Termite Populations

#### 2.1.1. Termite Identification

Termite soldier samples were stored and preserved in vials containing 90% ethanol. Termite soldier samples were placed under a dissecting microscope (Leica EZ24, Leica Microsystem, Singapore) and identified by examining the external morphology of the soldiers. Based on Thapa (1982) and Tho (1992), the shape and characteristics of the head and mandibles, antennae, and notum, size, and coloration of the termites were observed to identify the species of the samples [18,19].

#### 2.1.2. Study Site

**Figure 1:**
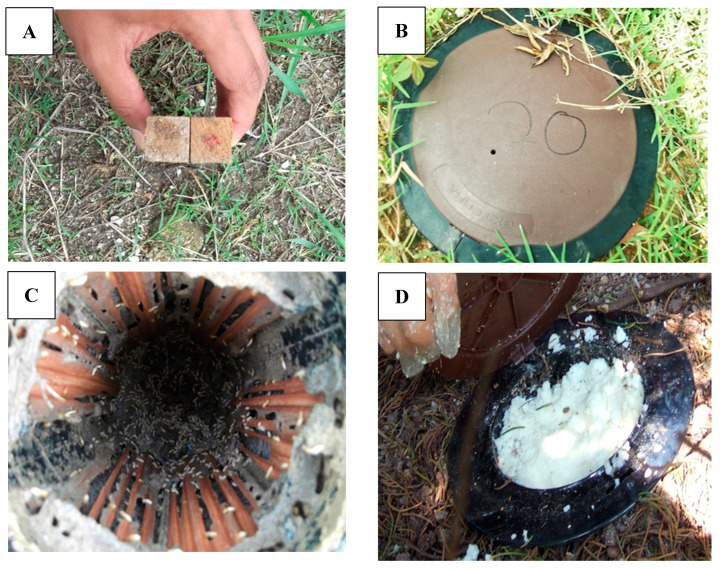
(**A**) Survey wooden stakes installed. (**B**) Exterra station replaced infested survey stake. (**C**) Active termite station with feeding consumption data recorded. (**D**) Only one active termite station was treated.

The experiment was carried out in Penang and Kedah, Malaysia. Five of the six sites were in Penang while one was in Kedah. Table 1 shows all of the locations involved in this study. Before the establishment of colony infestation status, oven-dried wooden stakes (2 cm diameter × 8 cm height), were installed surrounding the perimeter of the infested building (Figure 1A). The wooden stakes were buried at a depth of approximately 2.5 cm below the level of the ground. All wooden stakes that were attacked by the termites were replaced with Exterra monitoring stations (Ensystex, Malaysia Sdn. Bhd., Kuala Lumpur, Malaysia) (Figure 1B) and were labeled as active stations to record termite feeding activities (Figure 1C). Additional artificial underground monitoring stations were also placed next to the Exterra monitoring station to record termite feeding activities and were also labeled as active stations. Before the treatment, all active termite stations were inspected every two weeks to record the consumption data. At the end of the experiment, chlorfluazuron baits were placed in one of the active termite monitoring stations (Figure 1D).

#### 2.1.3. Bait Station Preparation

**Figure 2:**
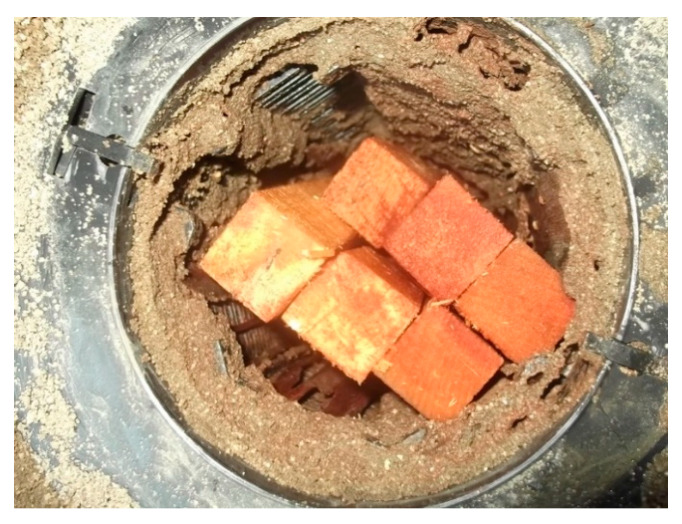
Wooden stakes were placed inside inground monitoring station (IGS).

Two types of termite collection devices (stations) were used to position the bait matrixes during the treatment: in-ground stations (IGSs) and above-ground stations (AGSs). An IGS is usually a 1.3 L cylindrical plastic container (Ensytex Malaysia Sdn. Bhd., Kuala Lumpur, Malaysia) with perforated sides and a hole at the bottom to allow termite ingress and egress. A termite interceptor wooden piece (*Eucalyptus delegatensis*) was mounted in the six inner walls of the IGSs to allow easy detection of termites and placement of the bait matrix without causing significant disruption to termite foraging activity in the station. Six wooden stakes (18 × 1.5 × 1.5 cm) of *Araucaria* sp. were also placed at the center of the IGSs to make the station more favorable to termites. The IGSs were installed around the infested building perimeter at intervals of 2 m and the number of IGSs installed in each infested building varied depending on the space available. The IGSs were then covered with soil to avoid any outside disturbances. Figure 2 shows the set-up of the IGSs.

**Figure 3:**
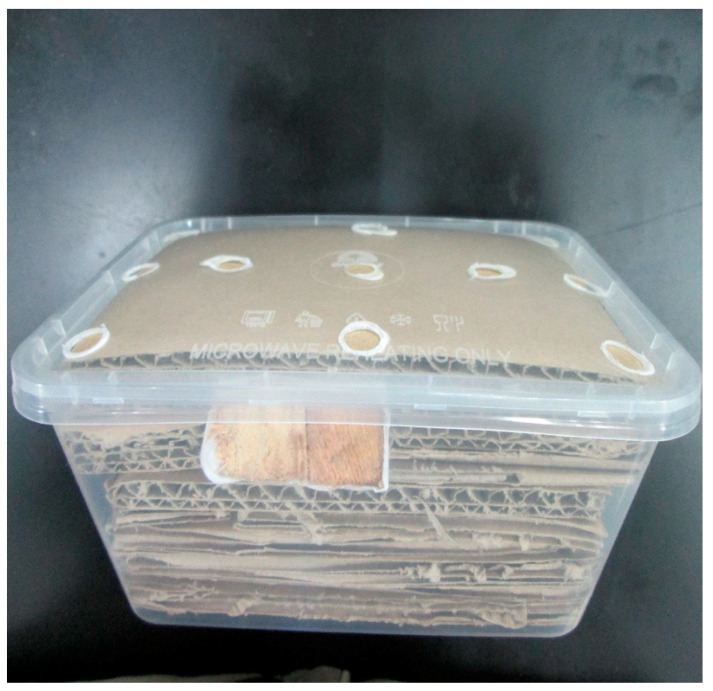
Corrugated cardboards and wooden stake were nicely arranged inside the above-ground stations (AGS).

The AGSs comprised plastic boxes with perforations on the bottom to allow the movement of termites in or out. Inside the AGSs, corrugated cardboard and a wooden stake (9 × 1.5 × 1.5 cm) were arranged and placed to enhance termite foraging activity. The stations were installed using adhesive tape or nails in areas where termite activity was located. The numbers of AGSs installed in each of the infested buildings varied depending on the number of areas of termite activity present. The AGSs were then covered with black plastic sheets to avoid any outside disturbances. Figure 3 shows how the AGSs were set up.

#### 2.1.4. Termite Feeding Consumption and Foraging Territory

At every two weeks, termites collected in the survey stakes inside IGSs and survey stakes inside monitoring stations and AGSs were brought back to the Household, Structural and Urban Entomology Laboratory, School of Biological Sciences, Universiti Sains Malaysia. In the laboratory, termites were separated by soil debris/corrugated cardboard using the method proposed by Tamashiro et al. (1973) [20]. Separated termites were then individually counted to obtain exact termite numbers (absolute calculations). Survey stakes and AGSs were washed/cleaned and oven-dried for 48 h. After 48 h, survey stakes and AGSs were weighed and compared to their weight before the installation to obtain the termite feeding consumption rate. The termite workers collected from a station with a heavy and active termite feeding activity were stained with 0.1% (wt/wt) Nile Blue A by a no choice feeding of Nile Blue A stained filter paper (Whatman No. 1, 9.0 cm in diameter) for 7 days [21,22]. The stained blue termites were released into the same station in which they were collected. At 14 days after the stained blue termites were released, the number of stained termites recaptured from the monitoring stations for that cycle was recorded [21,22]. This process was repeated for three rounds (triple mark recapture (TMR)). The termite colony foraging territory for each site was defined as the area encompassed by the stations containing stained blue termites during the TMR process [22,23].

#### 2.1.5. Bait Placement

**Figure 4:**
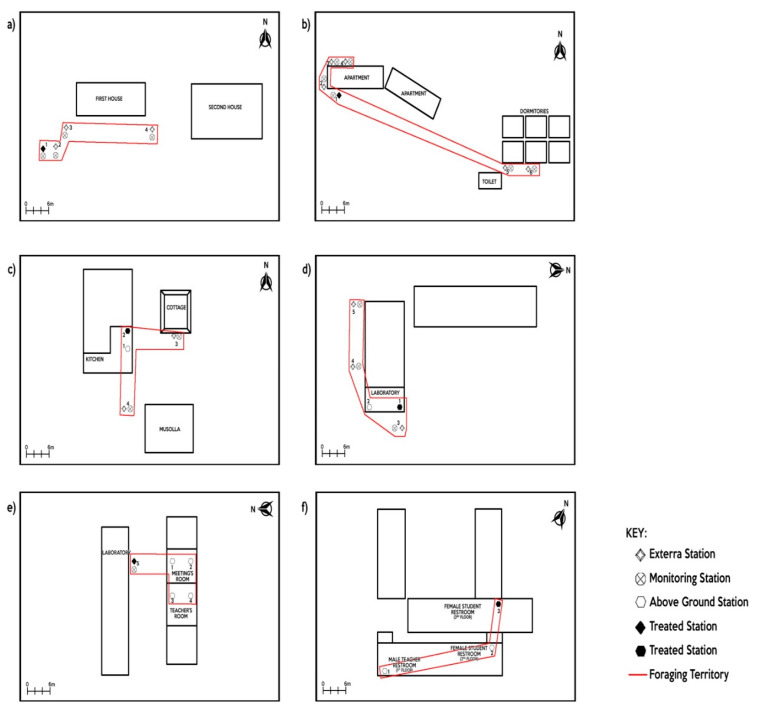
Selected treated station in (**a**) Bandar Baharu (BB), (**b**) CEMACS, (**c**) Taman Rupawan (TP), (**d**) SMK Bertam Perdana (SMKBP), (**e**) SMK Tuanku Puan Habsah (SMKTPH), (**f**) SMK Bukit Jambul (SMKBJH).

The IGSs/AGSs were inspected at intervals of two weeks. When termite activity was established, the stations were maintained to ensure that termite feeding activities continued. Maintenance was performed within the IGSs by cutting six wooden stakes (18 × 1.5 × 1.5 cm) and removing AGSs every two weeks. During the treatment, only one station was selected for bait placement at each of the sites. The selected station for baiting was chosen based on the active termite feeding consumption activities (wood consumption) within the termite foraging territories. If an IGS was chosen for bait placement, wooden stakes inside the IGSs were removed and replaced with doughy bait material (Requiem), a texture obtained by mixing the baits (100 g) with 400 mL of distilled water (1:4 wt:wt ratio). Similar to IGSs, corrugated cardboard and wooden stakes inside AGSs were removed and loaded with the same bait ratio (when AGS was chosen for bait placement) (Table 2). The bait was replenished at each inspection every two weeks until no termite feeding activity was detected at the bait stations. The treatment was considered successful when there were no longer termite feeding activities in all of the active IGSs and AGSs [24]. Figure 4a–f indicates the location of the selected station for the termite bait placement.

#### 2.1.6. Data Analysis

A *t*-test statistical analysis was conducted to evaluate the effectiveness of chlorfluazuron baits to eliminate a termite colony at each of the study sites. The total number of termites at day zero of the treatment was compared to the total termite number for the subsequent following two weeks until the whole colony was eliminated. The percentage of termite reduction was evaluated

## 3. Results

### 3.1. Evaluation of the Effectiveness of Selective Baiting Using Chlorfluazuron to Control Termite Populations

**Figure 5:**
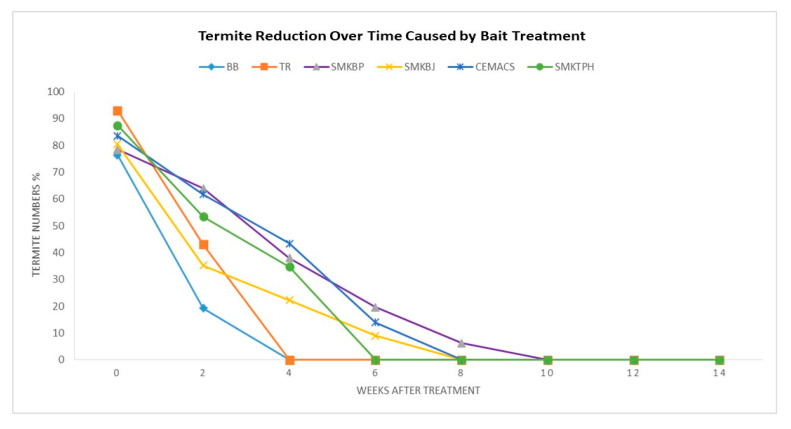
Fluctuation of total termite numbers after the baiting treatment applied. BB = Bandar Baharu; TR = Taman Rupawan; SMKBP = SMK Bertam Perdana; SMKBJ = SMK Bukit Jambul; CEMACS; SMKTPH = SMK Tunku Puan Habsah.

The termite species were identified as *Coptotermes gestroi* (Wasmann) at each of the six study sites. Figure 5 indicates the percentage of termite reduction from the active termite feeding station over time caused by bait treatment for colony elimination at each of the study sites. The details of the treatments are shown in Table 3.

#### 3.1.1. Bandar Baharu (BB)

This study found that the total number of termites at the end of treatment was statistically significantly lower (15.94 ± 30.6) compared to before treatment (62.57 ± 16.48), t (10) = 3.287, *p* = 0.008. There were four active in-ground stations in Bandar Baharu and all stations showed no post-treatment termite activity, indicating colony elimination took about four weeks. A total of 282.2 g of bait was consumed during the treatment. The number of termites decreased by 25% after two weeks of baiting. The termites were 100% eliminated at four weeks of treatment.

#### 3.1.2. CEMACS

This study found that the total number of termites at the end of treatment was statistically significantly lower (33.78 ± 34.73) compared to before treatment (88.67 ± 1.71), t (10) = 3.867, *p* = 0.003. There were six active in-ground stations and no above-ground stations in CEMACS, and all stations showed no termite activity after the treatment, which took approximately 8.6 weeks for colony eradication. A total of 296.7 g of bait was used to deliver the treatment. At week two of the treatment, 73.8% termite activity was observed. Termite activity was reduced to 51.86% in the following week of the treatment. At week six, activity was 16.72%, and was finally controlled at week eight of treatment. 

#### 3.1.3. Taman Rupawan (TR)

This study found that the total number of termites at the end of treatment was statistically significantly lower (7.18 ± 17.58) compared to before treatment (82.35 ± 10.98), t (10) = 8.885, *p* = 0.000. There were two active in-ground stations and two active above-ground stations in Taman Rupawan and all stations showed no termite activity after the treatment, which took approximately four weeks for colony termination. A total of 278.6 g of bait was consumed throughout the treatment. The number of termites was reduced by 46% after two weeks of baiting. At four weeks of treatment, the termites were 100% eliminated.

#### 3.1.4. SMK Bertam Perdana (SMKBP)

This study found that the total number of termites at the end of the treatment was statistically significantly lower in number (36.13 ± 29.67) than before treatment (86.35 ± 2.84), t (10) = 4.127, *p*= 0.002. There were three active in-ground stations and two active above-ground stations in SMK Bertam Perdana and all stations showed no termite activity after the treatment, which took approximately 8.6 weeks for colony elimination using a total of 283.7 g of bait. The number of termites was reduced to 81.5% after the first two weeks of baiting. Termite numbers were reduced to 48.4% at four weeks of treatment. At six weeks, the number of termites decreased to 28% and continued to decrease to 7.97% in week eight. At week 10, the termite colony was eventually eliminated.

#### 3.1.5. SMK Tunku Puan Habsah

This study found that the total number of termites at the end of treatment was statistically significantly lower (29.30 ± 36.28) compared to before treatment (90.80 ± 1.49), t (10) = 4.149, *p* = 0.002. There was one active in-ground station and four active above-ground stations in SMK Tunku Puan Habsah and all stations showed no termite activity after the treatment, which took approximately 6.3 weeks for colony elimination. A total of 216.4 g of bait was consumed by the termites to kill the entire population. Reduction of the number of termites resulted in 61.1% observed activity after two weeks of treatment. Activity was reduced to 39.7% at week four of the treatment and was finally eradicated at week six of the treatment.

#### 3.1.6. SMK Bukit Jambul

This study found that the total number of termites at the end of treatment was statistically significantly lower (25.57 ± 29.68) compared to before treatment (90.34 ± 2.23), t (10) = 5.332, *p* = 0.000. There were no active in-ground stations and three active above-ground stations in SMK Bukit Jambul and all stations had no termite activity after the treatment, which took approximately 8.6 weeks for colony termination with a total of 278.4 g of bait used for treatment. Two weeks after treatment, termite activity was observed at 43.7%. In week four, 27.8% remained, and 11.11% at week six. The treatment ended at week eight with no termites remaining.

## 4. Discussion

Termite populations were successfully eliminated using chlorfluazuron-containing alpha-cellulose powder as the active ingredient, which serves as an inhibitor of chitin-synthesis (CSI). However, there was no fixed time needed to suppress the termite colony. Bandar Baharu and Taman Rupawan took approximately four weeks for colony elimination, while three sites (SMK Bertam Perdana, SMK Bukit Jambul, and CEMACS) took approximately 8.6 weeks and SMK Tunku Puan Habsah took 6.3 weeks to eliminate all termite infestation. As a comparison, according to Peters et al. (2008), *Coptotermes. acinaciformis* and *C. frenchi* colonies in Australia were terminated within 16 weeks (112 days) when baited with chlorfluazuron bait, *C. curvignathus* in Malaysia eliminated within eight weeks (56 days) when baited with hexaflumuron bait, and *C. vastator* in The Philippines required eight weeks (56 days) when baited with chlorfluazuron bait [25]. Additionally, the mound-building termite, *C. acinaciformis,* requires 16 weeks to cause colony elimination when baited with chlorfluazuron bait [13]. The time differences needed for colony elimination vary according to several factors. According to Lee et al. (1999) and Sajap et al. (2000), the variations in the efficacy of these baits depends on how other termite species react to the baits; *Coptotermes* spp. have a higher feeding rate under tropical conditions in Malaysia [26,27]. Moreover, tropical termite species tend to have smaller population sizes and smaller foraging territories relative to temperate species [22,28]. In addition, the colony elimination also depends on the colony size and foraging territories [22]. Thus, this will lead to variation of time for colony elimination.

Another factor to be considered in the time difference needed to eliminate the colony is that the bait is toxicantly targeted toward species. *Coptotermes* and *Schedorhinotermes* are the most vulnerable species towards the tested bait [29]. In Malaysia, a *Coptotermes* colony requires a minimum of one month to suppress while the *Schedorhinotermes* species requires a minimum of six months [29]. By comparison, our results showed the termite colony baited at a single active termite station in Bandar Baharu and Taman Rupawan was eliminated in the first month, while the remaining sites took approximately 6.3–8.6 weeks to complete the elimination of termites. Higher termite genera such as *Macrotermes*, *Globitermes*, and *Microtermes* are less susceptible to the baits [29]. Chlorfluazuron is primarily successful in colony extinction of *Coptotermes* spp. while *Macrotermes gilvus*, *Odontotermes formosanus*, and *Globitermes sulphureus* consume more bait for a longer period to achieve colony elimination [30].

There are many advantages of choosing termite baiting treatment: (1) only a small amount of active ingredients (AIs) are used in a single treatment; (2) baits are mounted within the stations thus preventing any disturbance; (3) the AIs do not penetrate the water or soil; (4) baits are odorless; (5) no drills are needed; (6) no chemical residue remains after treatment; (7) in all cases, termite colonies were eliminated. Nonetheless, several risks need to be considered before opting for bait treatment: (1) a longer time is needed to take effect; (2) successful treatment can only occur when termites find and eat the baits; (3) no residue remains at the end of treatment to protect buildings; (4) the cost is greater than that of liquid termiticides [31]. Baiting is costly due to the cost of installing the baiting stations, and the expenses of the baits and a routine pest management professional (PMP) service to track the baiting operation [31]. 

Selected baited termite stations in this experiment would save time for a PMP as they only need to bait one of the active termite stations. Reducing the number of treated stations would also help minimize cost since less bait would be used, making this approach more cost-effective for clients. Thus, this approach may be significantly better than the conventional method in which the PMP treats all of the active termite stations because only selected termite stations would be handled using the presented approach, thereby ensuring savings in terms of inspection time and costs. Both methods can eliminate the colonies in the same amount of time.

## 5. Conclusions

Overall, this study found that a termite colony population can be eliminated by selective termite baiting treatment. Elimination of the termite population using selective termite baiting was successful with the aid of the termite behavior of trophallaxis, by which toxicants are shared among termite nestmates. This sharing of toxicant among nestmates circulates the active ingredients throughout the colony, thus eliminating the colony in the process. Regardless of the number of in-ground and above-ground stations, placing bait in just one of the active stations was sufficient to kill the colony because all of the interceptor and monitoring stations shared the same tunneling pathways leading to the colony. Chlorfluazuron baits needed an average of 4–8.6 weeks to eliminate termite infestation at the study sites.

## Figures and Tables

**Table 1:** Locations and description of study sites.

State	Sites	Latitude	Longitude
Kedah	Bandar Baharu (BB)	5°06′26″ N	100°32′30″ E
Penang	CEMACS	5°28′01″ N	100°12′00″ E
	Taman Rupawan (TR)	5°30′25″ N	100°26′34″ E
	SMK Bertam Perdana (SMKBP)	5°31′50″ N	100°27′59″ E
	SMK Tunku Puan Habsah (SMKTPH)	5°25′15″ N	100°18′27″ E
	SMK Bukit Jambul (SMKBJ)	5°20′54″ N	100°17′27″ E

**Table 2:** The total number of active stations at the study sites.

Sites	Number of Active Stations	Number of Above-Ground Stations (AGS)	Number of In-Ground Stations (IGS)	Number of Stations Baited
Bandar Baharu (BB)	4	0	4	1
CEMACS	6	0	6	1
Taman Rupawan (TP)	4	2	2	1
SMK Bertam Perdana (SMKBP)	5	2	3	1
SMK Tuanku Puan Habsah (SMKTPH)	5	4	1	1
SMK Bukit Jambul (SMKBJ)	3	3	0	1

**Table 3:** Summary of termite baiting at each of the study sites.

Sites	Total Number of Termites		Bait Consumed (g)	Time Taken for Colony Elimination (Weeks)
	Before	After		
Bandar Baharu (BB)	62.57 ± 16.48	15.94 ± 30.6	282.2	4
CEMACS	88.67 ± 1.71	33.78 ± 34.73	296.7	8.6
Taman Rupawan (TR)	82.35 ± 10.98	7.18 ± 17.58	278.6	4
SMK Bertam Perdana (SMKBP)	86.35 ± 2.84	36.13 ± 29.67	283.7	8.6
SMK Tunku Puan Habsah (SMKTPH)	90.80 ± 1.49	29.30 ± 36.28	216.4	6.3
SMK Bukit Jambul (SMKBJ)	90.34 ± 2.23	25.57 ± 29.68	278.4	8.6
